# Information Processing: The Language and Analytical Tools for Cognitive Psychology in the Information Age

**DOI:** 10.3389/fpsyg.2018.01270

**Published:** 2018-08-08

**Authors:** Aiping Xiong, Robert W. Proctor

**Affiliations:** Department of Psychological Sciences, Purdue University, West Lafayette, IN, United States

**Keywords:** cybernetics, small data, information age, information theory, scientific methods

## Abstract

The information age can be dated to the work of Norbert Wiener and Claude Shannon in the 1940s. Their work on cybernetics and information theory, and many subsequent developments, had a profound influence on reshaping the field of psychology from what it was prior to the 1950s. Contemporaneously, advances also occurred in experimental design and inferential statistical testing stemming from the work of Ronald Fisher, Jerzy Neyman, and Egon Pearson. These interdisciplinary advances from outside of psychology provided the conceptual and methodological tools for what is often called the cognitive revolution but is more accurately described as the information-processing revolution. Cybernetics set the stage with the idea that everything ranging from neurophysiological mechanisms to societal activities can be modeled as structured control systems with feedforward and feedback loops. Information theory offered a way to quantify entropy and information, and promoted theorizing in terms of information flow. Statistical theory provided means for making scientific inferences from the results of controlled experiments and for conceptualizing human decision making. With those three pillars, a cognitive psychology adapted to the information age evolved. The growth of technology in the information age has resulted in human lives being increasingly interweaved with the cyber environment, making cognitive psychology an essential part of interdisciplinary research on such interweaving. Continued engagement in interdisciplinary research at the forefront of technology development provides a chance for psychologists not only to refine their theories but also to play a major role in the advent of a new age of science.

Information is information, not matter or energy

Wiener (1952, p. 132)

## Introduction

The period of human history in which we live is frequently called the *information age*, and it is often dated to the work of Wiener (1894–1964) and Shannon (1916–2001) on cybernetics and information theory. Each of these individuals has been dubbed the “father of the information age” (Conway and Siegelman, 2005; Nahin, 2013). Wiener’s and Shannon’s work quantitatively described the fundamental phenomena of communication, and subsequent developments linked to that work had a profound influence on re-shaping many fields, including cognitive psychology from what it was prior to the 1950s (Cherry, 1957; Edwards, 1997, p. 222). Another closely related influence during that same period is the statistical hypothesis testing of Fisher (1890–1962), the father of modern statistics and experimental design (Dawkins, 2010), and Jerzy Neyman (1894–1981), and Egon Pearson (1895–1980). In the U.S., during the first half of the 20th century, the behaviorist approach dominated psychology (Mandler, 2007). In the 1950s, though, based mainly on the progress made in communication system engineering, as well as statistics, the human information-processing approach emerged in what is often called the cognitive revolution (Gardner, 1985; Miller, 2003).

The information age has had, and continues to have, a great impact on psychology and society at large. Since the 1950s, science and technology have progressed with each passing day. The promise of the information-processing approach was to bring knowledge of human mind to a level in which cognitive mechanisms could be modeled to explain the processes between people’s perception and action. This promise, though far from completely fulfilled, has been increasingly realized. However, as any period in human history, the information age will come to an end at some future time and be replaced by another age. We are not claiming that information will become obsolete in the new age, just that it will become necessary but not sufficient for understanding people and society in the new era. Comprehending *how* and *why* the information-processing revolution in psychology occurred should prepare psychologists to deal with the changes that accompany the new era.

In the present paper, we consider the information age from a historical viewpoint and examine its impact on the emergence of contemporary cognitive psychology. Our analysis of the historical origins of cognitive psychology reveals that applied research incorporating multiple disciplines provided conceptual and methodological tools that advanced the field. An implication, which we explore briefly, is that interdisciplinary research oriented toward solving applied problems is likely to be the source of the next advance in conceptual and methodological tools that will enable a new age of psychology. In the following sections, we examine milestones of the information age and link them to the specific language and methodology for conducting psychological studies. We illustrate how the research methods and theory evolved over time and provide hints for developing research tools in the next age for cognitive psychology.

## Cybernetics and Information Theory

### Wiener and Cybernetics

Norbert Wiener is an individual whose impact on the field of psychology has not been acknowledged adequately. Wiener, a mathematician and philosopher, was a child prodigy who earned his Ph.D. from Harvard University at age 18 years. He is best-known for establishing what he labeled *Cybernetics* (Wiener, 1948b), which is also known as control theory, although he made many other contributions of note. A key feature of Wiener’s intellectual development and scientific work is its interdisciplinary nature (Montagnini, 2017b).

Prior to college, Wiener was influenced by Harvard physiologist Walter B. Cannon (Conway and Siegelman, 2005), who later, in 1926, devised the term *homeostasis*, “the tendency of an organism or a cell to regulate its internal conditions, usually by a system of feedback controls…” (Biology Online Dictionary, 2018). During his undergraduate and graduate education, Wiener was inspired by several Harvard philosophers (Montagnini, 2017a), including William James (pragmatism), George Santayana (positivistic idealism), and Josiah Royce (idealism and the scientific method). Motivated by Royce, Wiener made his commitment to study logic and completed his dissertation on mathematic logic. Following graduate school, Wiener traveled on a postdoctoral fellowship to pursue his study of mathematics and logic, working with philosopher/logician Bertrand Russell and mathematician/geneticist Godfrey H. Hardy in England, mathematicians David Hilbert and Edmund Landau in Europe, and philosopher/psychologist John Dewey in the U.S.

Wiener’s career was characterized by a commitment to apply mathematics and logic to real-world problems, which was sparked by his working for the U.S. Army. According to Hulbert (2018, p. 50),

He returned to the United States in 1915 to figure out what he might do next, at 21 jumping among jobs… His stint in 1918 at the U.S. Army’s Aberdeen Proving Ground was especially rewarding…. Busy doing invaluable work on antiaircraft targeting with fellow mathematicians, he found the camaraderie and the independence he yearned for. Soon, in a now-flourishing postwar academic market for the brainiacs needed in a science-guided era, Norbert found his niche. At MIT, social graces and pedigrees didn’t count for much, and wartime technical experience like his did. He got hired. The latest mathematical tools were much in demand as electronic communication technology took off in the 1920s.

Wiener began his early research in applied mathematics on stochastic noise processes (i.e., Brownian motion; Wiener, 1921). The Wiener process named in honor of him has been widely used in engineering, finance, physical sciences, and, as described later, psychology. From the mid 1930s until 1953, Wiener also was actively involved in a series of interdisciplinary seminars and conferences with a group of researchers that included mathematicians (John von Neumann, Walter Pitts), engineers (Julian Bigelow, Claude Shannon), physiologists (Warren McCulloch, Arturo Rosenblueth), and psychologists (Wolfgang Köhler, Joseph C. R. Licklider, Duncan Luce). “Models of the human brain” is one topic discussed in those meetings, and concepts proposed during those conferences had significant influence on the research in information technologies and the human sciences (Heims, 1991).

One of Wiener’s major contributions was in World War II, when he applied mathematics to electronics problems and developed a statistical prediction method for fire control theory. This method predicted the position in space where an enemy aircraft would be located in the future so that an artillery shell fired from a distance would hit the aircraft (Conway and Siegelman, 2005). As told by Conway and Siegelman, “Wiener’s focus on a practical real-world problem had led him into that paradoxical realm of nature where there was no certainty, only probabilities, compromises, and statistical conclusions…” (p. 113). Advances in probability and statistics provided a tool for Wiener and others to investigate this paradoxial realm. Early in 1942 Wiener wrote a classified report for the National Defense Research Committee (NRDC), “The Extrapolation, Interpolating, and Smoothing of Stationary Time Series,” which was published as a book in 1949. This report is credited as the founding work in communications engineering, in which Wiener concluded that communication in all fields is in terms of information. In his words,

The proper field of communication engineering is far wider than that generally assigned to it. Communication engineering concerns itself with the transmission of messages. For the existence of a message, it is indeed essential that variable information be transmitted. The transmission of a single fixed item of information is of no communicative value. We must have a repertory of possible messages, and over this repertory a measure determining the probability of these messages (Wiener, 1949, p. 2).

Wiener went on to say “such information will generally be of a statistical nature” (p. 10).

From 1942 onward, Wiener developed his ideas of control theory more broadly in *Cybernetics*, as described in a *Scientific American* article (Wiener, 1948a):

It combines under one heading the study of what in a human context is sometimes loosely described as thinking and in engineering is known as control and communication. In other words, cybernetics attempts to find the common elements in the functioning of automatic machines and of the human nervous system, and to develop a theory which will cover the entire field of control and communication in machines and in living organisms (p. 14).

Wiener (1948a) made apparent in that article that the term cybernetics was chosen to emphasize the concept of *feedback* mechanism. The example he used was one of human action:

Suppose that I pick up a pencil. To do this I have to move certain muscles. Only an expert anatomist knows what all these muscles are, and even an anatomist could hardly perform the act by a conscious exertion of the will to contract each muscle concerned in succession. Actually, what we will is not to move individual muscles but to pick up the pencil. Once we have determined on this, the motion of the arm and hand proceeds in such a way that we may say that the amount by which the pencil is not yet picked up is decreased at each stage. This part of the action is not in full conscious (p. 14; see also p. 7 of Wiener, 1961).

Note that in this example, Wiener hits on the central idea behind contemporary theorizing in action selection – the choice of action is with reference to a distal goal (Hommel et al., 2001; Dignath et al., 2014). Wiener went on to say,

To perform an action in such a manner, there must be a report to the nervous system, conscious or unconscious, of the amount by which we have failed to pick up the pencil at each instant. The report may be visual, at least in part, but it is more generally kinesthetic, or, to use a term now in vogue, proprioceptive (p. 14; see also p. 7 of Wiener, 1961).

That is, Wiener emphasizes the role of negative feedback in control of the motor system, as in theories of motor control (Adams, 1971; Schmidt, 1975).

Wiener (1948b) developed his views more thoroughly and mathematically in his master work, *Cybernetics or Control and Communication in the Animal and in the Machine*, which was extended in a second edition published in 1961. In this book, Wiener devoted considerable coverage to psychological and sociological phenomena, emphasizing a systems view that takes into account feedback mechanisms. Although he was interested in sensory physiology and neural functioning, he later noted, “The need of including psychologists had indeed been obvious from the beginning. He who studies the nervous system cannot forget the mind, and he who studies the mind cannot forget the nervous system” (Wiener, 1961, p. 18).

Later in the *Cybernetics* book, Wiener indicated the value of viewing society as a control system, stating “Of all of these anti-homeostatic factors in society, the control of the means of communication is the most effective and most important” (p. 160). This statement is followed immediately by a focus on information processing of the individual: “One of the lessons of the present book is that any organism is held together in this action by the possession of means for the acquisition, use, retention, and transmission of information” (Wiener, 1961, p. 160).

Cybernetics, or the study of control and communication in machines and living things, is a general approach to understanding self-regulating systems. The basic unit of cybernetic control is the negative feedback loop, whose function is to reduce the sensed deviations from an expected outcome to maintain a steady state. Specifically, a present condition is perceived by the input function and then compared against a point of reference through a mechanism called a comparator. If there is a discrepancy between the present state and the reference value, an action is taken. This arrangement thus constitutes a closed loop of control, the overall purpose of which is to minimize deviations from the standard of comparison (reference point). Reference values are typically provided by superordinate systems, which output behaviors that constitute the setting of standards for the next lower level.

Cybernetics thus illustrates one of the most valuable characteristics of mathematics: to identify a common feature (feedback) across many domains and then study it abstracted from those domains. This abstracted study draws the domains closer together and often enables results from one domain to be extended to the other. From its birth, Wiener conceived of cybernetics as an interdisciplinary field, and control theory has had a major impact on diverse areas of work, such as biology, psychology, engineering, and computer science. Besides the mathematical nature, cybernetics has also been claimed as the science of complex probabilistic systems (Beer, 1959). In other words, cybernetics is a science of combined constant flows of communication and self-regulating systems.

### Shannon and Information Theory

With backgrounds in electrical engineering and mathematics, Claude Shannon obtained his Ph.D. in electrical engineering at MIT in 1940. Shannon is known within psychology primarily for information theory, but prior to his contribution on that topic, in his Master’s thesis, he showed how to design switch circuits according to Boole’s symbolic logic. Use of combinations of switches that represent binary values provides the foundation of modern computers and telecommunication systems (O’Regan, 2012). In the 1940s, Shannon’s work on digit circuit theory opened the doors for him and allowed him to make connections with great scientists of the day, including von Neumann, Albert Einstein, and Alan Turing. These connections, along with his work on cryptography, affected his thoughts about communication theory.

With regard to information theory, or what he called communication theory, Shannon (1948a) stated the essential problem of communication in the first page of his classic article:

The fundamental problem of communication is that of reproducing at one point either exactly or approximately a message selected at another point… The significant aspect is that the actual message is one selected from a set of possible messages. The system must be designed to operate for each possible selection, not just the one which will actually be chosen since this is unknown at the time of design. If the number of messages in the set is finite then this number or any monotonic function of this number can be regarded as a measure of the information produced when one message is chosen from the set, all choices being equally likely. As was pointed out by Hartley the most natural choice is the logarithmic function (p. 379).

**FIGURE 1:**
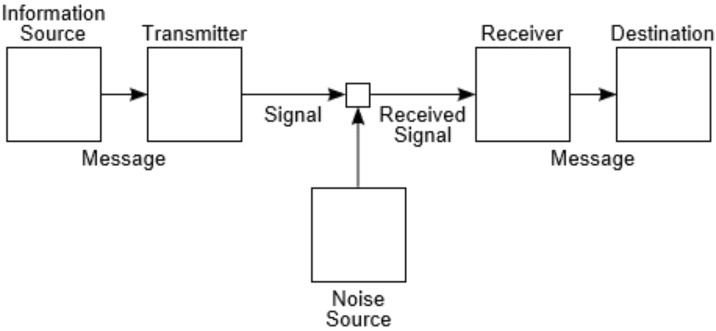
Shannon’s schematic diagram of a general communication system (Shannon, 1948a).

Shannon (1948a) characterized an information system as having five elements: (1) an information source; (2) a transmitter; (3) a channel; (4) a receiver; (5) a destination. Note the similarity of **Figure 1**, taken from his article, to the human information-processing models of cognitive psychology. Shannon provided mathematical analyses of each element for three categories of communication systems: discrete, continuous, and mixed. A key measure in information theory is *entropy*, which Shannon defined as the amount of uncertainty involved in the value of a random variable or the outcome of a random process. Shannon also introduced the concepts of *encoding* and *decoding* for the transmitter and receiver, respectively. His main concern was to find explicit methods, also called *codes*, to increase the efficiency and reduce the error rate during data communication over noisy channels to near the *channel capacity.*

Shannon explicitly acknowledged Wiener’s influence on the development of information theory:

Communication theory is heavily indebted to Wiener for much of its basic philosophy and theory. His classic NDRC report, *The Interpolation, Extrapolation, and Smoothing of Stationary Time Series* (Wiener, 1949), contains the first clear-cut formulation of communication theory as a statistical problem, the study of operations on time series. This work, although chiefly concerned with the linear prediction and filtering problem, is an important collateral reference in connection with the present paper. We may also refer here to Wiener’s *Cybernetics* (Wiener, 1948b), dealing with the general problems of communication and control (Shannon and Weaver, 1949, p. 85).

Although Shannon and Weaver (1949) developed similar measures of information independently from Wiener, they approached the same problem from different angles. Wiener developed the statistical theory of communication, equated information with negative entropy and related it to solve the problems of prediction and filtering while he worked on designing anti-aircraft fire-control systems (Galison, 1994). Shannon, working primarily on cryptography at Bell Labs, drew an analogy between a secrecy system and a noisy communication system through coding messages into signals to transmit information in the presence of noise (Shannon, 1945). According to Shannon, the amount of information and channel capacity were expressed in terms of positive entropy. With regard to the difference in sign for entropy in his and Wiener’s formulations, Shannon wrote to Wiener:

I do not believe this difference has any real significance but is due to our taking somewhat complementary views of information. I consider how much information is produced when a choice is made from a set – the larger the set the more the information. You consider the larger uncertainty in the case of a larger set to mean less knowledge of the situation and hence less information (Shannon, 1948b).

A key element of the mathematical theory of communication developed by Shannon is that it omits “the question of interpretation” (Shannon and Weaver, 1949). In other words, it separates information from the “psychological factors” involved in the ordinary use of information and establishes a neutral or non-specific human meaning of the information content (Luce, 2003). In such sense, consistent with cybernetics, information theory also confirmed this neutral meaning common to systems of machines, human beings, or combinations of them. The view that information refers not to “what” you send but what you “can” send, based on probability and statistics, opened a new science that used the same methods to study machines, humans, and their interactions.

## Inference Revolution

Although it is often overlooked, a related impact on psychological research during roughly the same period was that of using statistical thinking and methodology for small sample experiments. The two approaches that have been most influential in psychology, the null hypothesis significance testing of Ronald Fisher and the more general hypothesis testing view of Jerzy Neyman and Egon Pearson, resulted in what Gigerenzer and Murray (1987) called the *inference revolution*.

### Fisher, Information, Inferential Statistics, and Experiment Design

Ronald Fisher got his degree in mathematics from Cambridge University where he spent another year studying statistical mechanics and quantum theory (Yates and Mather, 1963). He has been described as “a genius who almost single-handedly created the foundations for modern statistical science” (Hald, 2008, p. 147) and “the single most important figure of 20th century statistics” (Efron, 1998, p. 95). Fisher is also “rightly regarded as the founder of the modern methods of design and analysis of experiments” (Yates, 1964, p. 307). In addition to his work on statistics and experimental design, Fisher made significant scientic contributions to genetics and evolutionary biology. Indeed, Dawkins (2010), the famous biologist, called Fisher the greatest biologist since Darwin, saying:

Not only was he the most original and constructive of the architects of the neo-Darwinian synthesis. Fisher also was the father of modern statistics and experimental design. He therefore could be said to have provided researchers in biology and medicine with their most important research tools.

Our interest in this paper is, of course, with the research tools and logic that Fisher provided, along with their application to scientific content.

Fisher began his early research as a statistician at Rothamsted Experimental Station in Harpenden, England (1919–1933). There, he was hired to develop statistical methods that could be applied to interpret the cumulative results of agriculture experiments (Russell, 1966, p. 326). Besides dealing with the past data, he became involved with ongoing experiments and developing methods to improve them (Lehmann, 2011). Fisher’s hands-on work with experiments is a necessary feature of his background for understanding his positions regarding statistics and experimental design. Fisher (1962, p. 529) essentially said as much in an address published posthumously:

There is, frankly, no easy substitute for the educational discipline of whole time personal responsibility for the planning and conduct of experiments, designed for the ascertainment of fact, or the improvement of Natural Knowledge. I say “educational discipline” because such experience trains the mind and deepens the judgment for innumerable ancillary decisions, on which the value or cogency of an experimental program depends.

The analysis of variance (ANOVA, Fisher, 1925) and an emphasis on experimental design (Fisher, 1937) were both outcomes of Fisher’s work in response to the experimental problems posed by the agricultural research performed at Rothamsted (Parolini, 2015).

Fisher’s work synthesized mathematics with practicality and reshaped the scientific tools and practice for conducting and analyzing experiments. In the preface to the first edition of his textbook *Statistical Methods for Research Workers*, Fisher (1925, p. vii) made clear that his main concern was application:

Daily contact with the statistical problems which present themselves to the laboratory worker has stimulated the purely mathematical researches upon which are based the methods here presented. Little experience is sufficient to show that the traditional machinery of statistical processes is wholly unsuited to the needs of practical research.

Although prior statisticians developed probabilistic methods to estimate errors of experimental data [e.g., Student’s, 1908 (Gosset’s) *t*-test], Fisher carried the work a step further, developing the concept of null hypothesis testing using ANOVA (Fisher, 1925, 1935). Fisher demonstrated that by proposing a null hypothesis (usually no effect of an independent variable over a population), a researcher could evaluate whether a difference between conditions was sufficiently unlikely to occur due to chance to allow rejection of the null hypothesis. Fisher proposed that tests of significance with a low *p*-value can be taken as evidence against the null hypothesis. The following quote from Fisher (1937, pp. 15–16), captures his position well:

It is usual and convenient for experimenters to take 5 per cent. as a standard level of significance, in the sense that they are prepared to ignore all results which fail to reach this standard, and, by this means, to eliminate from further discussion the greater part of the fluctuations which chance causes have introduced into their experimental results.

While Fisher recommended using the 0.05 probability level as a criterion to decide whether to reject the null hypothesis, his general position was that researchers should set the critical level of significance at sufficiently low probability so as to limit the chance of concluding that an independent variable has an effect when the null hypothesis is true. Therefore, the criterion of significance does not necessarily have to be 0.05 (see also Lehmann, 1993), and Fisher’s main point was that failing to reject the null hypothesis regardless of what criterion is used does not warrant accepting it (see Fisher, 1956, pp. 4 and 42).

Fisher (1925, 1935) also showed how different sources of variance can be partitioned to allow tests of separate and combined effects of two or more independent variables. Prior to this work, much experimental research in psychology, though not all, used designs in which a single independent variable was manipulated. Fisher made a case that designs with two or more independent variables were more informative than multiple experiments using different single independent variables because they allowed determination of whether the variables interacted. Fisher’s application of the ANOVA to data from factorial experimental designs, coupled with his treatment of extracting always the maximum amount of information (likelihood) conveyed by a statistic (see later), apparently influenced both Wiener and Shannon (Wiener, 1948a, p. 10).

In “The Place of Design of Experiments in the Logic of Scientific Inference,” Fisher (1962) linked experimental design, the application of correct statistical methods, and the subsequent extraction of a valid conclusion through the concept of information (known as *Fisher information*). Fisher information measures the amount of information that an obtained random sample of data has about the variable of interest (Ly et al., 2017). It is the expected value of the second moment of the log-likelihood function, which is the probability density function for obtained data conditional on the variable. In other words, the variance is defined to be the Fisher information, which measures the sensitivity of the likelihood function to the changes of a manipulated variable on the obtained results. Furthermore, Fisher argued that experimenters should be interested in not only minimizing loss of information in the process of statistical reduction (e.g., use ANOVA to summarize evidence that preserves the relevant information from data, Fisher, 1925, pp. 1 and 7) but also the deliberate study of experimental design, for example, by introducing randomization or control, to maximize the amount of information provided by estimates derived from the resulting experimental data (see Fisher, 1947). Therefore, Fisher unfied experimental design and statistical analysis through information (Seidenfeld, 1992; Aldrich, 2007), an approach that resonates with the system view of cybernetics.

### Neyman-Pearson Approach

Jerzy Neyman obtained his doctorate from the University of Warsaw with a thesis based on his statistical work at the Agricultural Institute in Bydgoszcz, Poland, in 1924. Egon Pearson received his undergraduate degree in mathematics and continued his graduate study in astronomy at Cambridge University until 1921. In 1926, Neyman and Pearson started their collaboration and raised a question with regard to Fisher’s method, why test only the null hypothesis? They proposed a solution in which not only the null hypothesis but also a class of possible alternatives are considered, and the decision is one of accepting or rejecting the null hypothesis. This decision yields probabilities of two kinds of error: false rejection of the null hypothesis (Type I or Alpha) or false acceptance of the alternative hypothesis (Type II or Beta; Neyman and Pearson, 1928, 1933). They suggested that the best test was the one that minimized the Type II error subject to a bound on the Type I error, i.e., the significance level of the test. Thus, instead of classifying the null hypothesis as rejected or not, the central consideration of the Neyman-Pearson approach was that one must specify not only the null hypothesis but also the alternative hypotheses against which it is tested. With this symmetric decision approach, statistical power (1 – Type II error) becomes an issue. Fisher (1947) also realized the importance and necessity of power but argued that it is a qualitative concern addressed during the experimental design to increase the sensitivity of the experiment and not part of the statistical decision process. In other words, Fisher thought that researchers should “conduct experimental and observational inquiries so as to maximize the information obtained for a given expenditure” (Fisher, 1951, p. 54), but did not see that as being part of statistics.

To reject or accept the null hypothesis, rather than disregarding results that do not allow rejection of the null hypothesis, was the rule of behavior for Neyman-Pearson hypothesis testing. Thus, their approach assumed that a decision made from the statistical analysis was sufficient to draw a conclusion as to whether the null or alternative hypothesis was most likely and did not put emphasis on the need for non-statistical inductive inference to understand the problems. In other words, tests of significance were interpreted as means to make decisions in an acceptance procedure (also see Wald, 1950) but not specifically for research workers to gain a better understanding of the experimental material. Also, the Neyman-Pearson approach interpreted probability (or the value of a significance level) as a realization of long-run frequency in a sequence of repetitions under constant conditions. Their view was that if a sequence of independent events was obtained with probability *p* of success, then the long-run success frequency will be close to *p* (this is known as a frequentist probability, Neyman, 1977). Fisher (1956) vehemently disagreed with this frequentist position.

### Fisherian vs. Frequentist Approaches

In a nutshell, the differences between Fisherians and frequentists are mostly about research philosophy and how to interpret the results (Fisher, 1956). In particular, Fisher emphasized that in scientific research, failure to reject the null hypothesis should not be interpreted as acceptance of it, whereas Neyman and Pearson portrayed the process as a decision between accepting or rejecting the null hypothesis. Nevertheless, the usual practice for statistical testing in psychology is based on a hybrid of Fisher’s and Neyman-Pearson’s approaches (Gigerenzer and Murray, 1987). In practice, when behavioral researchers speak of the results of research, they are primarily referring to the statistically significant results and less often to null effects and the effect size estimates associated with those *p*-values.

The reliance on the results of significance testing has been explained from two perspectives: (1) Neither experienced behavioral researchers nor experienced statisticians have a good intuitive feel for the practical meaning of effect size estimation (e.g., Rosenthal and Rubin, 1982); (2) The reliance on a reject-or-accept dichotomous decision procedure, in which the differences between *p* levels are taken to be trivial relative to the difference between exceeding or failing to exceed a 0.05 or some other accepted level of significance (Nelson et al., 1986). The reject-accept procedure follows the Neyman-Pearson approach and is compatible with the view that information is binary (Shannon, 1948a). Nevertheless, even if an accurate statistical power analysis is conducted, a replication study properly powered can produce results that are consistent with the effect size of interest or consistent with absolutely no effect (Nelson et al., 1986; Maxwell et al., 2015). Therefore, instead of solely relying on hypothesis testing, or whether an effect is true or false, a report of actual *p* level obtained along with a statement of the effect size estimation, should be considered.

Fisher emphasized in his writings that an essential ingredient in the research process is the judgment of the researcher, who must decide by how much the obtained results have advanced a particular theoretical proposition (that is, how meaningful the results are). This decision is based in large part on decisions made during experimental design. The statistical significance test is just a useful tool to inform such decisions during the process to allow the researcher to be confident that the results are likely not due to chance. Moreover, he wanted this statistical decision in scientific research to be independent of a priori probabilities or estimates because he did not think these could be made accurately. Consequently, Fisher considered that only a statistically significant effect in an exact test for which the null hypothesis can be rejected should be open to subsequent interpretation by the researcher.

Acree (1978, pp. 397–398) conducted a thorough evaluation of statistical inference in psychological research that for the most part captures why Fisher’s views had greater impact on the practice of psychological researchers than those of Neyman and Pearson (emphasis ours):

On logical grounds, Neyman and Pearson had decidedly the better theory; but Fisher’s claims were *closer to the ostensible needs of psychological research*. The upshot is that psychologists have mostly followed Fisher in their thinking and practice: in the use of the hypothetical infinite population to justify probabilistic statements about a single data set; in treating the significance level evidentially; in setting it after the experiment is performed; in never accepting the null hypothesis; in disregarding power…. Yet the rationale for all our statistical methods, insofar as it is presented, is that of Neyman and Pearson, rather than Fisher.

Although Neyman and Pearson may have had “decidedly the better theory” for statistical decisions in general, Fisher’s approach provides a better theory for scientific inferences from controlled experiments.

## Interim Summary

The work we described in Sections “Cybernetics and Information Theory” and “Inference Revolution” identifies three crucial pillars of research that were developed mainly in the period from 1940 to 1955: cybernetics/control theory, information/communication theory, and inferential statistical theory. Moreover, our analysis revealed a correspondence among those pillars. Specifically, the cybernetics/control theory corresponds to the experimental design, both of which provide the framework for cognitive psychology. The information theory corresponds to the statistical test, both of which provide quantitative evidence for the qualitative assumption.

These pillars were identified as early as 1952 in the preface to the proceedings of a conference called *Information Theory in Biology*, in which the editor, Quastler (1953, p. 1), said:

The “new movement” [what we would call information-processing theory] is based on evaluative concepts (R. A. Fisher’s experimental design, A. Wald’s statistical decision function, J. von Neumann’s theory of games), on the development of a measure of information (R. Hartley, D. Gabor, N. Wiener, C. Shannon), on studies of control mechanisms, and the analysis and design of large systems (W. S. McCulloch and W. Pitt’s “neurons,” J. von Neumann’s theory of complicated automata, N. Wiener’s cybernetics).

The pillars undergirded not only the new movement in biology but also the new movement in psychology. The concepts introduced in the dawning information age of 1940–1955 had tremendous impact on applied and basic research in experimental psychology that transformed psychological research into a form that has developed to the present.

## Human Information Processing

As noted in earlier sections, psychologists and neurophysiologists were involved in the cybernetics, information theory, and inferential statistics movements from the earliest days. Each of these movements was crucial to the ascension of the information-processing approach in psychology and the emergence of cognitive science, which are often dated to 1956. In this section, we review developments in cognitive psychology linked to each of the three pillars, starting with the most fundamental one, cybernetics.

### The Systems Viewpoint of Cybernetics

George A. Miller explicitly credited cybernetics as being seminal in 1979, stating, “I have picked September 11, 1956 [the date of the second MIT symposium on Information Theory] as the birthday of cognitive science – the day that cognitive science *burst from the womb of cybernetics* and became a recognizable interdisciplinary adventure in its own right” (quoted by Elias, 1994, p. 24; emphasis ours). With regard to the development of human factors (ergonomics) in the United Kingdom, Waterson (2011, pp. 1126–1127) remarks similarly:

During the 1960s, the ‘systems approach’ within ergonomics took on a precedence which has lasted until the present day, and a lot of research was informed from cybernetics and general systems theory. In many respects, a concern in applying a systemic approach to ergonomic issues could be said to be one of the factors which ‘glues’ together all of the elements and sub-disciplines within ergonomics.

This seminal role for cybernetics is due to its fundamental idea that various levels of processing in humans and non-humans can be viewed as control systems with interconnected stages and feedback loops. It should be apparent that the human information-processing approach, which emphasizes the human as a processing system with feedback loops, stems directly from cybernetics, and the human-machine system view that underlies contemporary human factors and ergonomics, can be traced directly to cybernetics.

We will provide a few more specific examples of the impact of cybernetics. McCulloch and Pitts (1943), members of the cybernetics movement, are given credit for developing “the first conceptual model of an artificial neural network” (Shiffman, 2012) and “the first modern computational theory of mind and brain” (Piccinini, 2004). The McCulloch and Pitts model identified the neurons as logical decision elements by on and off states, which are the basis of building brain-like machines. Since then, Boolean function, together with feedback through neurons, has been used extensively to quantify theorizing in relation to both neural and artificial intelligent systems (Piccinini, 2004). Thus, computational modeling of brain processes was part of the cybernetics movement from the outset.

Franklin Taylor a noted engineering psychologist, reviewed Wiener’ (1948b) *Cybernetics* book, calling it “a curious and provocative book” (Taylor, 1949, p. 236). Taylor noted, “The author’s most important assertion for psychology is his suggestion, often repeated, that computers, servos, and other machines may profitably be used as models of human and animal behavior” (p. 236), and “It seems that Dr. Wiener is suggesting that psychologists should borrow the theory and mathematics worked out for machines and apply them to the behavior of men” (p. 237). Psychologists have clearly followed this suggestion, making ample use of the theory and mathematics of control systems. Craik (1947, 1948) in the UK had in fact already started to take a control theory approach to human tracking performance, stating that his analysis “puts the human operator in the class of ‘intermittent definite correction servos’ ” (Craik, 1948, p. 148).

Wiener’s work seemingly had considerable impact on Taylor, as reflected in the opening paragraphs of a famous article by Birmingham and Taylor (1954) on human performance of tracking tasks and design of manual control systems:

The cardinal purpose of this report is to discuss a principle of control system design based upon considerations of engineering psychology. This principle will be found to advocate design practices for man-operated systems similar to those customarily employed by engineers with fully automatic systems…. In many control systems the human acts as the error detector… During the last decade it has become clear that, in order to develop control systems with maximum precision and stability, human response characteristics have to be taken into account. Accordingly, the new discipline of engineering psychology was created to undertake the study of man from an engineering point of view (p. 1748).

Control theory continues to provide a quantitative means for modeling basic and applied human performance (Jagacinski and Flach, 2003; Flach et al., 2015).

Colin Cherry, who performed the formative study on auditory selective attention, studied with Wiener and Jerome Wiesner at MIT in 1952. It was during this time that he conducted his classic experiments on the *cocktail party problem* – the question of how we identify what one person is saying when others are speaking at the same time (Cherry, 1953). His detailed investigations of selective listening, including attention switching, provided the basis for much research on the topic in the next decade that laid the foundation for contemporary studies of attention. The initial models explored the features and locus of a “limited-capacity processing channel” (Broadbent, 1958; Deutsch and Deutsch, 1963). Subsequent landmark studies of attention include the attentuation theory of Treisman (1960; also see Moray, 1959); capacity models that conceive of attention as a resource to be flexibly allocated to various stages of human information processing (Kahneman, 1973; Posner, 1978); the distinction between controlled and automatic processing (Shiffrin and Schneider, 1977); the feature-integration theory of visual search (Treisman and Gelade, 1980).

As noted, Miller (2003) and others identified the year 1956 as a critical one in the development of contemporary psychology (Newell and Simon, 1972; Mandler, 2007). Mandler lists two events that year that ignited the field, in both of which Allan Newell and Herbert Simon participated. The first is the meeting of the Special Group on Information Theory of the Institute of Electrical and Electronics Engineers, which included papers by linguist Noam Chomsky (who argued against an information theory approach to language for his transformational-generative grammer) and psychologist Miller (on avoiding the short-term memory bottleneck), in addition to Newell and Simon (on their Logic Theorist “thinking machine”) and others (Miller, 2003). The other event is the Dartmouth Summer Seminar on Artificial Intelligence (AI), which was organized by John McCarthy, who had coined the term AI the previous year. It included Shannon, Oliver Selfridge (who discussed initial ideas that led to his Pandemonium model of human pattern recognition, described in the next paragraph), and Marvin Minsky (a pioneer of AI, who turned to symbolic AI after earlier work on neural net; Moor, 2006), among others. A presentation by Newell and Simon at that seminar is regarded as essential in the birth of AI, and their work on human problem solving exploited concepts from work on AI.

Newell applied a combination of experimental and theoretical research during his work in RAND Corporation from 1950 (Simon, 1997). For example, in 1952, he and his colleagues designed and conducted laboratory experiments on a full-scale simulation of an Air-Force Early Warning Station to study the decision-making and information-handling processes of the station crews. Central to the research was the recording and analyzing the crew’s interaction with their radar screens, with interception aircraft, and with each other. From these studies, Newell became to believe that information processing is the central activity in organizations (systems).

Selfridge (1959) laid the foundation for a cognitive theory of letter perception with his Pandemonium model, in which the letter identification is achieved by way of hierarchically organized layers of features and letter detectors. Inspired by Selfridge’s work on Pandemonium, Newell started to converge on the idea that systems can be created that contain intelligence and have the ability to adapt. Based on his understanding of computers, heuristics, information processing in organizations (systems), and cybernetics, Newell (1955) delineated the design of a computer program to play chess in “The Chess Machine: An Example of Dealing with a Complex Task by Adaptation.” After that, for Newell, the investigation of organizations (systems) became the examination of the mind, and he committed himself to understand human learning and thinking through computer simulations.

In the study of problem solving, think-aloud protocols in laboratory settings revealed that means-end analysis is a key heuristic mechanism. Specifically, the current situation is compared to the desired goal state and mental or physical actions are taken to reduce the gap. Newell, Simon, and Jeff Shaw developed the General Problem Solver, a computer program that could solve problems in various domains if given a problem space (domain representation), possible actions to move between space states, and information about which actions would reduce the gap between the current and goal states (see Ernst and Newell, 1969, for a detailed treatment, and Newell and Simon, 1972, for an overview). The program built into the system underlined the importance of control structure for solving the problems, revealing a combination of cybernetics and information theory.

Besides using cybernetics, neuroscientists further developed it to explain anticipation in biological systems. Although closed-loop feedback can perform online corrections in a determinate machine, it does not give any direction (Ashby, 1956, pp. 224–225). Therefore, a feedforward loop was proposed in cybernetics that could improve control over systems through anticipation of future actions (Ashby, 1956; MacKay, 1956). Generally, the feedforward mechansim is constructed as another input pathway parallel to the actual input, which enables comparison between the actual and anticipated inputs before they are processed by the system (Ashby, 1960; Pribram, 1976, p. 309). In other words, a self-organized system is not only capable of self-adjusting its own behavior (feedback), but is also able to change its own internal organization in such a way as to select the response that eliminates a disturbance from the outside among the random responses that it attempts (Ashby, 1960). Therefore, the feedforward loop “nudges” the inputs based on predefined parameters in an automatic manner to account for cognitive adaptation, indicating a higher level action planning. Moreover, different from error-based feedback control, the knowledge-based feedforward control cannot be further adjusted once the feedforward input has been processed. The feedforward control from cybernetics has been used by psychologists to understand human action control at behavioral, motoric, and neural levels (for a review, see Basso and Olivetti Belardinelli, 2006).

Therefore, both feedback and feedforward are critical to a control system, in which feedforward control is valuable and could improve the performance when feedback control is not sufficient. A control system with feedforward and feedback loops allows the interaction between top-down and bottom-up information processing. Consequently, the main function of a control system is not to create “behavior” but to create and maintain the anticipation of a specific desired condition, which constitutes its reference value or standard of comparison.

Cognitive psychology and neuroscience suggest that the combination of anticipatory and hierarchical structures are involved for human action learning and control (Herbort et al., 2005). Specifically, anticipatory mechanisms lead to direct action selections in inverse model and effective filtering mechanisms in forward models, both of which are based on sensorimotor contingencies through people’s interaction with the environment (ideomotor principle, Greenwald, 1970; James, 1890). Therefore, the feedback loop included in cybernetics as well as the feedforward loop are essential to the learning processes.

We conclude this section with mention of one of the milestone books in cognitive psychology, *Plans and the Structure of Behavior*, by Miller et al. (1960). In the prolog to the book, the authors indicate that they worked on it together for a year at the Center for Advanced Study in the Behavioral Sciences in California. As indicated by the title, the central idea motivating the book was that of a plan, or program, that guides behavior. But, the authors said:

Our fundamental concern, however, was to discover whether the *cybernetic ideas* have any relevance for psychology…. There must be some way to phrase the new ideas [of cybernetics] so that they can contribute to and profit from the science of behavior that psychologists have created. It was the search for that favorable intersection that directed the course of our year-long debate (p. 3, emphasis ours).

In developing the central concept of the test-operate-test (TOTE) unit in the book, Miller et al. (1960) stated, “The interpretation to which the argument builds is one that has been called the ‘cybernetic hypothesis,’ namely that the fundamental building block of the nervous system is the feedback loop” (pp. 26–27). As noted by Edwards (1997), the TOTE concept “is the same principle upon which Weiner, Rosenblueth, and Bigelow had based ‘Behavior, Purpose, and Teleology”’ (p. 231). Thus, although Miller later gave 1956 as the date that cognitive science “burst from the womb of cybernetics,” even after the birth of cognitive science, the genes inherited from cybernetics continued to influence its development.

### Information and Uncertainty

Information theory, a useful way to quantify psychological and behavior concepts, had possibly a more direct impact than cybernetics on psychological research. No articles were retrieved from the PsycINFO database prior to 1950 when we entered “information theory” as an unrestricted field search term on May 3, 2018. But, from 1950 to 1956 there were 37 entries with “information theory” in the title and 153 entries with the term in some field. Two articles applying information theory to speech communication appeared in 1950, a general theoretical article by Fano (1950) of the Research Laboratory in Electronics at MIT, and an empirical article by Licklider (1950) of the Acoustics Laboratory, also at MIT. Licklider (1950) presented two methods of reducing the frequncies of speech without destoying the intelligibility by using the Shannon-Weaver information formula based on first-order probability.

Given Licklider’s background in cybernetics and information theory, it is not too surprising that he played a major role in establishing the ARPAnet, which was later replaced by the Internet:

His 1968 paper called “The Computer as a Communication Device” illustrated his vision of network applications and predicted the use of computer networks for communications. Until then, computers had generally been thought of as mathematical devices for speeding up computations (Internet Hall of Fame, 2016).

Licklider worked from 1943–1950 at the Psyco-Acoustics Laboratory (PAL) of Harvard University University, headed by Edwards (1997, p. 212) noted, “The PAL played a crucial role in the genesis of postwar information processing psychologies.” He pointed out, “A large number of those who worked at the lab… helped to develop computer models and metaphors and to introduce information theory into human experimental psychology” (p. 212). Among those were George Miller and Wendell Garner, who did much to promulgate information theory in psychology (Garner and Hake, 1951; Miller, 1953), as well as Licklider, Galanter and Pribram. Much of PAL’s research was based in solving engineering problems for the military and industry.

The exploration of human information-processing limitations using information theory that led to one of the most influential applications was that of Hick (1952) and Hyman (1953) to explain increases in reaction time as a function of uncertainty regarding the potential stimulus-response alternatives. Their analyses showed that reaction time increased as a logarithmic function of the number of equally likely alternatives and as a function of the amount of information as computed from differential probabilities of occurrence and sequential effects. This relation, call Hick’s law or the Hick-Hyman law has continued to be a source of research to the present and is considered to be a fundamental law of human-computer interaction (Proctor and Schneider, 2018). Fitts and Seeger (1953) showed that uncertainty was not the only factor influencing reaction time. They examined performance of eight-choice task for all combinations of three spatial-location stimulus and response arrays. Responses were faster and more accurate when the response array corresponded to that of the stimulus array than when it did not, which Fitts and Seeger called a stimulus-response compatibility effect. The main point of their demonstration was that correspondence of the spatial codes for the stimulus and response alternatives was crucial, and this led to detailed investigation of compatibility effects that continue to the present (Proctor and Vu, 2006, 2016).

Even more influential has been Fitts’s law, which describes movement time in tasks where people make discrete aimed movements to targets or series of repetitive movements between two targets. Fitts (1954) developed the index of difficulty as –log_2_ W/2A bits/response, where *W*, is the target width and *A* is the amplitude (or distance) of the movement. The resulting movement time is a linear function of the index of difficulty, with the slope differing for different movement types. Fitts’s law continues to be the subject of basic and applied research to the present (Glazebrook et al., 2015; Velasco et al., 2017).

Information theory was applied to a range of other topics during the 1950s, including intelligence tests (Hick, 1951), memory (Aborn and Rubenstein, 1952; Miller, 1956), perception (Attneave, 1959), skilled performance (Kay, 1957), music (Meyer, 1957), and psychiatry (Brosin, 1953). However, the key concept of information theory, entropy, or uncertainty, was found not to provide an adequate basis for theories of human performance (e.g., Ambler et al., 1977; Proctor and Schneider, 2018).

Shannon’ (1948a) advocacy of information theory for electronic communication was mainly built on there being a mature understanding of the structured pattern of information transmission within electromagnetic systems at that time. In spite of cognitive research having greatly expanded our knowledge about how humans select, store, manipulate, recover, and output information, the fundamental mechanisms of those information processes remained under further investigation (Neisser, 1967, p. 8). Thus, although information theory provided a useful mathematic metric, it did not provide a comprehensive account of events between the stimulus and response, which is what most psychologists were interested in (Broadbent, 1959). With the requirement that information be applicable to a vast array of psychological issues, “information” has been expanded from a measure of informativeness of stimuli and responses, to a framework for describing the mental or neural events between stimuli and responses in cognitive psychology (Collins, 2007). Therefore, the more enduring impact of information theory was through getting cognitive psychologists to focus on the nature of human information processing, such that by Lachman et al. (1979) titled their introduction to the field, *Cognitive Psychology and Information Processing*.

Along with the information theory, the arrival of the computer provided one of the most viable models to help researchers understand the human mind. Computers grew from a desire to make machines smart (Laird et al., 1987), which assumes that stored knowledge inside of a machine can be applied to the world similar to the way that people do, constituting intelligence (e.g., intelligent machine, Turing, 1937; AI, Minsky, 1968; McCarthy et al., 2006). The core idea of the computer metaphor is that the mind functions like a digital computer, in which mental states are computational states and mental processes are computational processes. The use of the computer as a tool for thinking about how the mind handles information has been highly influential in cognitive psychology. For example, the PsycINFO database returned no articles prior to 1950 when “encoding” was entered as an unrestricted field search term on May 3, 2018. From 1950 to 1956 there was 1 entry with “encoding” in the title and 4 entries with the term in some field. But, from 1956 to 1973, there were 214 entries with “encoding” in the title and 578 entries with term in some field, including the famous encoding specificity principle of Tulving and Thomson (1973). Some models in cognitive psychology were directly inspired by how the memory system of a computer works, for example, the multi-store memory (Atkinson and Shiffrin, 1968) and working memory (Baddeley and Hitch, 1974) models.

Although cybernetics is the origin of early AI (Kline, 2011) and the computer metaphor and cybernetics share similar concepts (e.g., representation), they are fundamentally different at the conceptual level. The computer metaphor represents a genuine simplification: Terms like “encoding” and “retrieving” can be used to describe human behavior analogously to machine operation but without specifying a precise mapping between the analogical “computer” and the target “human” domain (Gentner and Grudin, 1985). In contrast, cybernetics provides a powerful framework to help people understand the human mind, which holds that, regardless of human or machine, it is necessary and possible to achieve goals through correcting action using feedback and adapting to the external environment using feedforward. Recent breakthroughs in AI (e.g., AlphaGo beating professional Go players) rely on training the machine to learn how to perform tasks at a level not seen before using a large number of examples and an artificial neural network (ANN) without human guidance. This unsupervised learning allows the machine to determine on its own whether a certain function should be executed. The development of ANN has been greatly influenced by consideration of dynamic properties of cybernetics (Cruse, 2009), to achieve the goal of self-organization or self-regulation.

### Statistical Inference and Decisions

Statistical decision theory also had substantial impact. Engineering psychologists were among the leaders in promulgating use of the ANOVA, with Chapanis and Schachter (1945; Schachter and Chapanis, 1945) using it in research on depth perception through distorted glass, conducted in the latter part of World War II and presented in Technical Reports. As noted by Rucci and Tweney (1980), “Following the war, these [engineering] psychologists entered the academic world and began to publish in regular journals, using ANOVA” (p. 180).

Factorial experiments and use of the ANOVA were slow to take hold in psychology. Rucci and Tweney (1980) counted the frequency with which the *t*-test and ANOVA were used in major psychology journals from 1935 to 1952. They described the relation as, “Use of both *t* and ANOVA increased gradually prior to World War II, declined during the war, and increased immediately thereafter” (p. 172). Rucci and Tweney concluded, “By 1952 it [ANOVA] was fully established as the most frequently used technique in experimental research” (p. 166). They emphasized that this increased use of ANOVA reflected a radical change in experimental design, and emphasized that although one could argue that the statistical technique caused the change in psychological research, “It is just as plausible that the discipline had developed in such a way that the time was ripe for adoption of the technique” (p. 167). Note that the rise in use of null hypothesis testing and ANOVA paralleled that of cybernetics and information theory, which suggests that the time was indeed ripe for the use of probability theory, multiple independent variables, and formal scientific decision making through hypothesis testing that is embodied in the factorial design and ANOVA.

The first half of the 1950s also saw the introduction of signal detection theory, a variant of statistical decision theory, for analyzing human perception and performance. Initial articles by Peterson et al. (1954) and Van Meter and Middleton (1954) were published in a journal of the Institute of Electrical and Electronics Engineers (IEEE), but psychologists were quick to realize the importance of the approach. This point is evident in the first sentence of Swets et al.’s (1961) article describing signal detection theory in detail:

About 5 years ago, the theory of statistical decision was translated into a theory of signal detection. Although the translation was motivated by problems in radar, the detection theory that resulted is a general theory… The generality of the theory suggested to us that it might also be relevant to the detection of signals by human observers… The detection theory seemed to provide a framework for a realistic description of the behavior of the human observer in a variety of perceptual tasks (p. 301).

Signal detection theory has proved to be an invaluable tool because it dissociates influences of the evidence on which decisions are based from the criteria applied to that evidence. This way of conceiving decisions is useful not only for perceptual tasks but for a variety of tasks in which choices on the basis of noisy information are required, including recognition memory (Kellen et al., 2012). Indeed, Wixted (2014) states, “Signal-detection theory is one of psychology’s most notable achievements, but it is not a theory about typical psychological phenomena such as memory, attention, vision or psychopathology (even though it applies to all of those areas and more). Instead, it is a theory about how we use evidence to make decisions.”

In the 1960s, Sternberg (1969) formalized the additive factors method of analyzing reaction-time data to identify different information-processing stages. Specifically, a factorial experiment is conducted, and if two independent variables affect different processing stages, the two variables do not interact. If, on the other hand, there is a significant interaction, then the variables can be assumed to affect at least one processing stage in common. Note that the subtitle of Sternberg’s article is “Extension of Donders’ Method,” which is reference to the research reported by F. C. Donders 100 years earlier in which he estimated the time for various processing stages by subtracting the reaction time obtained for a task that did not have an additional processing stage inserted from one that did. A limitation of Donders’s (1868/1969) subtraction method is that the stages had to be assumed and could not be identified. Sternberg’s extension that provided a means for identifying the stages did not occur until both the language of information processing and the factorial ANOVA were available as tools for analyzing reaction-time data. Additive factors method formed a cornerstone for much research in cognitive psychology for the following couple of decades, and the logic is still often applied to interpret empirical results, often without explicit acknowledgment.

In psychology, how people make decisions in perceptual and cognitive tasks has often been proposed on the basis of sequential sampling to explain the pattern of obtained reaction time (RT) and percentage error. The study of such mechanisms addresses one of the fundamental question in psychology, namely, how the central nervous system translates perception into action and how this translation depends on the interaction and expectation of individuals. Like signal detection theory, the theory of sequential sampling starts from the premise that perceptual and cognitive decisions are statistical in nature. It also follws the widely accepted assumption that sensory and cognitive systems are inherently noise and time-varying. In practice, the study of a given sequential model reduce to the study of a stochastic process, which represents the accumulative information avaliable to the decision at a given time. A Wiener process forms the basis of Ratcliff’ (1978) influential diffusion model of reaction times, in which noisy information accumulates continuously over time from a starting point to response thresholds (Ratcliff and Smith, 2004). Recently, Srivastava et al. (2017) extended this diffusion model to make the Wiener process time dependent. More generally, Shalizi (2007, p. 126) makes the point, “The fully general theory of stochastic calculus considers integration with respect to a very broad range of stochastic processes, but the original case, which is still the most important, is integration with respect to the Wiener process.”

In parallel to use of the computer metaphor to understand human mind, use of the laws of probability as metaphors of the mind also has had a profound influence on physiology and psychology (Gigerenzer, 1991). Gregory (1968) regarded seeing an object from an image as an inference from a hypothesis (also see “unconscious inference” of Helmholtz, 1866/1925). According to Gregory (1980), in spite of differences between perception and science, the cognitive procedures carried out by perceptual neural processes are essentially the same as the processes of predictive hypotheses of science. Especially, Gregory emphasized the importance and distinction between bottom–up and top–down procedures in perception. For normal perception and the perceptual illusions, the bottom–up procedures filter and structure the input and the top–down procedures refer to stored knowledge or assumptions that can work downwards to parcel signals and data into object.

A recent development of the statistics metaphor is the Bayesian brain hypothesis, which has been used to model perception and decision making since the 1990s (Friston, 2012). Rao and Ballard (1997) described a hierarchical neural network model of visual recognition, in which both input-driven bottom–up signals and expectation-driven top–down signals were used to predict the current recognition state. They showed that feedback from a higher layer to the input later carries predictions of expected inputs, and the feedforward connections convey the errors in prediction which are used to correct the estimation. Rao (2004) illustrated how the Bayesian model could be implemented with neural networks by feedforward and recurrent connections, showing that for both perception and decision-making tasks the resulting network exhibits direction selectivity and computes posterior error corrections.

We would like to highlight that, different from the analogy to computers, the cybernetics view is essential for the Bayesian brain hypothesis. This reliance on cybernetics is because the Bayesian brain hypothesis models the interaction between prior knowledge (top–down) and sensory evidence (bottom–up) quantitatively. Therefore, the success of Bayesian brain modeling is due to both the framework from cybernetics and the computation of probability. Seth (2015) explicitly acknowledges this relation in his article, *The Cybernetic Bayesian Brain*.

Meanwhile, the external information format (or representation) on which the Bayesian inferences and statistical reasoning operate has been investigated. For example, Gigerenzer and Hoffrage (1995) varied mathematically equivalent representation of information in percentage or frequency for various problems (e.g., the mammography problem, the cab problem) and found that frequency formats enabled participants’ inferences to conform to Bayes’ theorem without any teaching or instruction.

## Conclusion

The *Information Age* of humans follows on periods that are called the *Stone Age*, *Bronze Age*, *Iron Age*, and *Industrial Age*. These labels indicate that the advancement and time period of a specific human history are often represented by the type of tool material used by humans. The formation of the *Information Age* is inseparable from the interdisciplinary work of cybernetics, information theory, and statistical inference, which together generated a cognitive psychology adapted to the age. Each of these three pillars has been acknowledged separately by other authors, and contemporary scientifical approaches to motor control and cognitive processing have been continuously inspired by cybernetics, information theory, and statistical inference.

Kline (2015, p. 1) aptly summarized the importance of cybernetics in the founding of the information age:

During contentious meetings filled with brilliant arguments, rambling digressions, and disciplinary posturing, the cybernetics group shaped a language of feedback, control, and information that transformed the idiom of the biological and social sciences, sparked the invention of information technologies, and set the intellectual foundation for what came to be called the information age. The premise of cybernetics was a powerful analogy: that the principles of information-feedback machines, which explained how a thermostat controlled a household furnace, for example, could also explain how all living things—from the level of the cell to that of society—behaved as they interacted with their environment.

Pezzulo and Cisek (2016) extended the cybernetic principles of feedback and forward control for understanding cognition. In particular, they proposed hierachical feedback control, indicating that adaptive action selection is influenced not only by prediction of immediate outcomes but also prediction of new opportunites afforded by the outcomes. Scott (2016) highlighted the use of sensory feedback, after a person becomes familiar with performing a perceptual-motor task, to drive goal-directed motor control, reducing the role of top–down control through utilizing bottom–up sensory feedback.

Although less expansive than Kline (2015), Fan (2014, p. 2) emphasized the role of information theory. He stated, “Information theory has a long and distinguished role in cognitive science and neuroscience. The ‘cognitive revolution’ of the 1950s, as spearheaded by Miller (1956) and Broadbent (1958), was highly influenced by information theory.” Likewise, Gigerenzer (1991, p. 255) said, “Inferential statistics… provided a large part of the new concepts for mental processes that have fueled the so called cognitive revolution since the 1960s.” The separate treatment of the three pillars by various authors indicates that the pillars have distinct emphases, which are sometimes treated as in opposition (Verschure, 2016). However, we have highlighted the convergent aspects of the three that were critical to the founding of cognitive psychology and its continued development to the present. An example of a contemporary approach utilizing information theory and statistics in computational and cognitive neuroscience is the study of activity of neuronal populations to understand how the brain processes information. Quian Quiroga and Panzeri (2009) reviewed methods based on statistical decoding and information theory, and concluded, “Decoding and information theory describe complementary aspects of knowledge extraction… A more systematic joint application of both methodologies may offer additional insights” (p. 183).

Leahey (1992) claimed that it is incorrect to say that there was a “cognitive revolution” in the 1950s, but he acknowledged “that information-processing psychology has had world-wide influence…” (p. 315). Likewise, Mandler (2007) pointed out that the term “cognitive revolution” for the changes that occurred in the 1950s is a misnomer because, although behaviorism was dominant in the United States, much psychology outside of the United States prior to that time could be classified as “cognitive.” However, he also said, after reviewing the 1956 meetings and ones in 1958, that “the 1950s surely were ready for the emergence of the new information-processing psychology—the new cognitive psychology” (p. 187). Ironically, although both Leahey and Mandler identified the change as being one of information processing, neither author acknowledged the implication of their analyses, which is that there was a transformation that is more aptly labeled the information-processing revolution rather than the cognitive revolution. The concepts provided by the advances in cybernetics, information theory, and inferential statistical theory together provided the language and methodological tools that enabled a significant leap forward in theorizing.

Wootton (2015), says of his book *The Invention of Science: A New History of the Scientific Revolution*, “We can state one of its core premises quite simply: a revolution in ideas requires a revolution in language” (p. 48). That language is what the concepts of communications systems engineering and inferential statistical theory provided for psychological research. Assessing the early influence of cybernetics and information theory on cognitive psychology, Broadbent (1959) stated, “It is in fact, the cybernetic approach above all others which has provided a clear language for discussing those various internal complexities which make the nervous system differ from a simple channel” (p. 113). He also identified a central feature of information theory as being crucial: The information conveyed by a stimulus is dependent on the stimuli that might have occurred but did not.

Posner (1986), in his introduction to the Information Processing section of the *Handbook of Perception and Human Performance*, highlights more generally that the language of information processing affords many benefits to cognitive psychologists. He states, “Information processing language provides an alternative way of discussing internal mental operations intermediate between subjective experience and activity of neurons” (p. V-3). Later in the chapter, he elaborates:

The view of the nervous system in terms of information flow provided a common language in which both conscious and unconscious events might be discussed. Computers could be programmed to simulate exciting tasks heretofore only performed by human beings without requiring any discussion of consciousness. By analogies with computing systems, one could deal with the format (code) in which information is presented to the senses and the computations required to change code (recodings) and for storage and overt responses. These concepts brought a new unity to areas of psychology and a way of translating between psychological and physiological processes. The presence of the new information processing metaphor reawakened interest in internal mental processes beyond that of simple sensory and motor events and brought cognition back to a position of centrality in psychology (p. V-7).

Posner also notes, “The information processing approach has a long and respected relationship with applications of experimental psychology to industrial and military settings” (V-7). The reason, as emphasized years earlier by Wiener, is that it allows descriptions of humans to be integrated with those of the nonhuman parts of the system. Again, from our perspective, there was a revolution, but it was specifically an information-processing revolution.

Our main points can be summarized as follows:

(1) The information age originated in interdisciplinary research of an applied nature.
(2) Cybernetics and information theory played pivotal roles, with the former being more fundamental than the latter through its emphasis on a systems approach.
(3) These roles of communication systems theory were closely linked to developments in inferential statistical theory and applications.
(4) The three pillars of cybernetics, information theory, and inferential statistical theory undergirded the so-called cognitive revolution in psychology, which is more appropriately called the information-processing revolution.
(5) Those pillars, rooted in solving real-world problems, provided the language and methodological tools that enabled growth of the basic and applied fields of psychology.
(6) The experimental design and inferential statistics adopted in cognitive psychology, with emphasis on rejecting null hypotheses, originated in the applied statistical analyses of the scientist Ronald Fisher and were influential because of their compatibility with scientific research conducted using controlled experiments.

Simon (1969), in an article entitled “Designing Organizations for an Information-Rich World,” pointed out the problems created by the wealth of information:

Now, when we speak of an information-rich world, we may expect, analogically, that the wealth of information means a dearth of something else – a scarcity of whatever it is that information consumes. What information consumes is rather obvious: it consumes the attention of its recipients. Hence a wealth of information creates a poverty of attention, and a need to allocate that attention efficiently among the overabundance of information sources that might consume it (manuscript pp. 6-7).

What Simon described is valid and even more evident in the current smart device and Internet era, where the amount of information is overwhelming. Phenomena as disparate as accidents caused by talking on a cellphone while driving (Banducci et al., 2016) and difficulty assessing the credibility of information reported on the Internet or other media (Chen et al., 2015) can be attributed to the overload. Moreover, the rapid rate at which information is encountered may have a negative impact on maintaining a prolonged focus of attention (Microsoft Canada, 2015). Therefore, knowing how people process information and allocate attention is increasingly essential in the current explosion of information.

As noted, the predominant method of cognitive psychology in the information age has been that of drawing theoretical inferences from the statistical results of small-scale sets of data collected in controlled experimental settings (e.g., laboratory). The progress in psychology is tied to progress in statistics as well as technological developments that improve our ability to measure and analyze human behavior. Outside of the lab, with the continuing development of the Internet of Things (IoT), especially the implementation of AI, human physical lives are becoming increasingly interweaved into the cyber world. Ubiquitous records of human behavior, or “big data,” offer the potential to examine cognitive mechanisms at an escalated scale and level of ecological validity that cannot be achieved in the lab. This opportunity seems to require another significant transformation of cognitive psychology to use those data effectively to push forward understanding of the human mind and ensure seamless integration with cyber physical systems.

In a posthumous article, Brunswik (1956) noted that psychology should have the goal of broadening perception and learning by including interactions with a probabilistic environment. He insisted that psychology “must link behavior and environment statistically in bivariate or multivariate correlation rather than with the predominant emphasis on strict law…” (p. 158). As part of this proposal, Brunswik indicated a need to relate psychology more closely to disciplines that “use autocorrelation and intercorrelation, as theoretically stressed especially by Wiener (1949), for probability prediction” (p. 160). With the ubiquitous data being collected within cyber physical systems, more extensive use of sophisticated correlational methods to extract the information embedded within the data will likely be necessary.

Using Stokes (1997) two dimensions of scientific research (considerations of use; quest for fundamental understanding), the work of pioneers of the Information Age, including Wiener, Shannon, and Fisher, falls within *Pasteur’s Quadrant* of use-inspired basic research. They were motivated by the need to solve immediate applied problems and through their research advanced human’s fundamental understanding of nature. Likewise, in seizing the opportunity to use big data to inform cognitive psychology, psychologists need to increase their involvement in interdisciplinary research targeted at real-world problems. In seeking to mine the information from big data, a new age is likely to emerge for cognitive psychology and related disciplines.

Although we reviewed the history of the information-processing revolution and subsequent developments in this paper, our ultimate concern is with the future of cognitive psychology. So, it is fitting to end as we began with a quote from Wiener (1951, p. 68):

To respect the future, we must be aware of the past.

## Author Contributions

AX and RP contributed jointly and equally to the paper.

## Conflict of Interest Statement

The authors declare that the research was conducted in the absence of any commercial or financial relationships that could be construed as a potential conflict of interest.
